# Deprescribing and Polypharmacy for Medicare Beneficiaries Under Guardianship in Long-Term Care Facilities

**DOI:** 10.1093/geroni/igaa057.274

**Published:** 2020-12-16

**Authors:** Tami Swenson

**Affiliations:** Des Moines University, Des Moines, Iowa, United States

## Abstract

Local judicial courts vary in the amount of supervision they provide guardians, which makes the practice of guardianship uneven. To begin to address the evidence gap to inform best practices for persons under guardianship care, this study examines the issues of polypharmacy and prescribing patterns for four therapeutic classes most commonly targeted for deprescribing for older adults. The Medicare Current Beneficiary Survey (MCBS) for 2015 and 2016 is used to examine facility-dwelling Medicare beneficiaries under guardianship compared with those that are not. Logistic regression is used to examine association of polypharmacy outcomes and guardianship care controlling for patient and facility characteristics. Statistical models are adjusted using Fay’s Method with replicate weights for the MCBS complex survey design. Approximately 12% of the facility-dwelling Medicare population in 2015 and 2016 are persons under guardianship care. Persons under guardianship were more likely to have polypharmacy or to be prescribed 5 or more medications (Odd Ratio (OR)=1.168, 95% Confidence Interval (CI)=1.156 to 1.180, p<0.001) than facility-dwelling Medicare beneficiaries not under guardianship care. Medicare beneficiaries under guardianship were more likely to be prescribed PPIs (OR=1.229, 95% CI=1.222 to 1.237, p<0.001) or antipsychotic medications (OR=1.240, 95% CI=1.232 to 1.247, p<0.001) but less likely to be prescribed benzodiazepines (OR=0.920, 95% CI=0.913 to 0.927, p<0.001) or antihyperglycemics (OR-0.726, 95% CI=0.721 to 0.731, p<0.001). Medical decision support services, such as guardianship care, are increasing in importance as shared decision making between patients and physicians evolves to address polypharmacy and deprescribing for older adults.

